# Chimeric Cell Therapies as a Novel Approach for Duchenne Muscular Dystrophy (DMD) and Muscle Regeneration

**DOI:** 10.3390/biom14050575

**Published:** 2024-05-13

**Authors:** Katarzyna Budzynska, Maria Siemionow, Katarzyna Stawarz, Lucile Chambily, Krzysztof Siemionow

**Affiliations:** 1 Department of Orthopaedics, University of Illinois at Chicago, Chicago, IL 60607, USA; kat.budzynska@gmail.com (K.B.); kasiaworek1@gmail.com (K.S.); lucile.chambily@gmail.com (L.C.); siemiok@gmail.com (K.S.); 2 Chair and Department of Traumatology, Orthopaedics, and Surgery of the Hand, Poznan University of Medical Sciences, 61-545 Poznan, Poland

**Keywords:** cellular therapy, chimeric cells, chimerism, donor-recipient chimeric cells, Dystrophin Expressing Chimeric (DEC) cells, DEC therapy, Duchenne Muscular Dystrophy, muscle regeneration, stem cells

## Abstract

Chimerism-based strategies represent a pioneering concept which has led to groundbreaking advancements in regenerative medicine and transplantation. This new approach offers therapeutic potential for the treatment of various diseases, including inherited disorders. The ongoing studies on chimeric cells prompted the development of Dystrophin-Expressing Chimeric (DEC) cells which were introduced as a potential therapy for Duchenne Muscular Dystrophy (DMD). DMD is a genetic condition that leads to premature death in adolescent boys and remains incurable with current methods. DEC therapy, created via the fusion of human myoblasts derived from normal and DMD-affected donors, has proven to be safe and efficacious when tested in experimental models of DMD after systemic–intraosseous administration. These studies confirmed increased dystrophin expression, which correlated with functional and morphological improvements in DMD-affected muscles, including cardiac, respiratory, and skeletal muscles. Furthermore, the application of DEC therapy in a clinical study confirmed its long-term safety and efficacy in DMD patients. This review summarizes the development of chimeric cell technology tested in preclinical models and clinical studies, highlighting the potential of DEC therapy in muscle regeneration and repair, and introduces chimeric cell-based therapies as a promising, novel approach for muscle regeneration and the treatment of DMD and other neuromuscular disorders.

## 1. Introduction

The concept of chimerism originated from Greek mythology, where the Chimera was portrayed as a creature with the head of a lion, body of a goat, and the tail of a snake [1]. The idea of a single organism of two genetically distinct origins coexisting in harmony has evolved over the years, leading to the development of chimeric cells. Initial studies focused on bone marrow allotransplantation and provided a foundation for understanding the therapeutic benefits of chimerism [2,3,4,5,6,7]. Specifically, the potential for inducing tolerance has raised hopes for the use of chimeric cells in transplantation as a promising approach that could reduce the need for lifelong immunosuppression [8,9,10,11,12]. However, the recent evolution of chimerism-based studies led to significant advancements in the field and the broader application of chimeric cells in other areas, including regenerative medicine and the management of complex genetic disorders [5,6,7,13].

**Figure 1:**
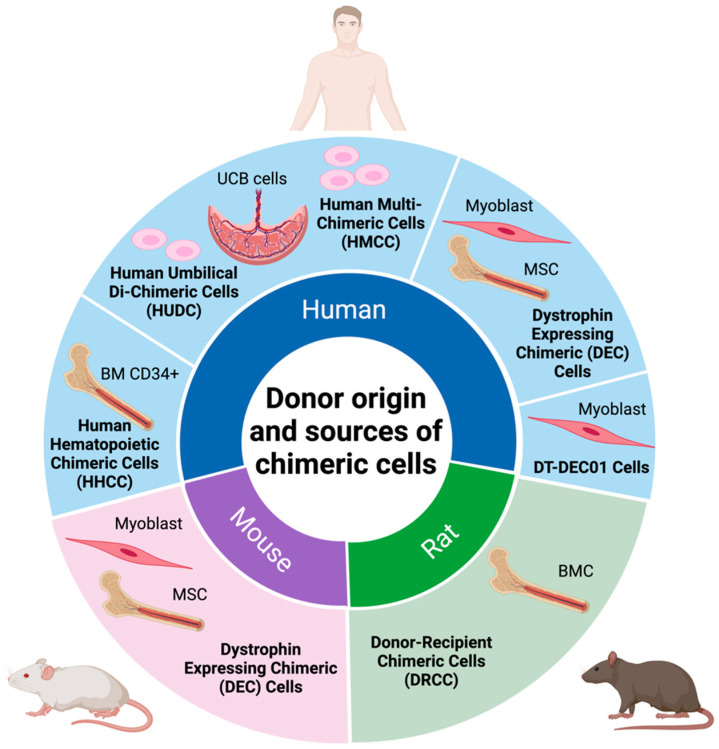
Summary of donor cells’ origins and sources for creation of chimeric cells. Various cell lineages, including myoblasts, bone marrow, and umbilical cord blood derived from different donors of human, rat, and mouse origins, were used for creation of the distinct chimeric cell lines. Figure created with BioRender.com.

In recent years, a variety of different cell lineages have been tested for the creation of chimeric cells to further explore their potential mechanisms and therapeutic efficacy. Research studies conducted by Siemionow’s team initially focused on the tolerance-inducing properties of the chimeric cells, promoting their use in vascularized composite allograft (VCA) transplantation [14,15]. The encouraging results of these studies led to the development of donor–recipient chimeric cell (DRCC) therapy, created by the fusion of bone marrow cells (BMC) derived from MHC-mismatched ACI (RT1^a^) and Lewis (RT1^1^) rats [8]. The application of DRCC in the VCA experimental model confirmed chimerism induction and extended VCA survival after the intraosseous administration of DRCC therapy [16]. The long-term engraftment and tolerogenic properties observed in VCA, following bone marrow transplantation (BMT), inspired the development of new chimeric cell lines, based on the hematopoietic cells, mesenchymal stem cells, and myoblasts [17,18,19,20] of different donor origin (Figure 1), such as the human hematopoietic chimeric cell (HHCC) line from BM-derived CD34^+^ cells [19] and human umbilical di-chimeric cells (HUDC) [20] originating from two unrelated human donors.

The concept of DRCC was applied for the creation of Dystrophin Expressing Chimeric (DEC) cell therapy as a novel approach for the treatment of Duchenne Muscular Dystrophy (DMD) [17,18,21]. The purpose of introducing DEC therapy was to promote muscle regeneration, reduce muscle pathology, and restore dystrophin expression in the organs most severely affected by DMD in order to improve muscle function. To ensure the distribution of DEC cells to *mdx* muscles, DEC therapy was initially created by the fusion of different variations of myoblast and mesenchymal stem cell lines to test their efficacy and long-term engraftment. The most promising outcomes were observed after the administration of DEC therapy based on the myoblasts derived from normal and DMD-affected donors. Following DEC administration, increased dystrophin expression was confirmed and correlated with functional and morphological improvements after administration to the *mdx* mouse model of DMD. Therefore, this DEC cell line was considered the most beneficial for further application in DMD patients.

DMD is a lethal genetic disorder associated with the X chromosome, and therefore, predominantly affects males [22,23]. The pathogenesis of the disease is linked to the alterations in the dystrophin gene. Dystrophin is a large protein crucial for stabilizing the sarcolemma of muscle fibers. Therefore, the malfunction of dystrophin leads to the progressive damage of muscle fibers, resulting in muscle wasting and weakness as healthy tissue is replaced by inflammatory changes and fibrosis [24,25]. The first symptoms of DMD typically appear between the ages of two and five, marked by delayed motor development, difficulty walking, and a characteristic Gower’s sign [26,27]. The condition progresses over time, inevitably leading to cardiorespiratory failure, which is the most common cause of death among DMD patients [28,29].

Despite the ongoing research focused on discovering breakthrough therapies that could provide a potential cure, DMD remains an untreatable disease. Current therapeutic approaches, including stem cell-based therapies, have been limited by challenges in cell engraftment and immune rejection [30,31,32,33,34]. Therefore, there is a great need for novel strategies that could offer treatments for DMD and other neuromuscular disorders.

This review article is based on the thorough literature search, mainly written in English, in the PubMed database, and explores the innovative application of chimeric cells, highlighting the role of DEC cells in promoting muscle regeneration and halting the disease’s progression. It outlines the safety, efficacy, and potential of chimeric cell therapies in the treatment of DMD, from the initial preclinical models to the first in-human study. This review underscores the promising future of chimeric cell therapies, not only in improving the quality of life for DMD patients, but also in pioneering a path toward novel therapeutic strategies for other diseases associated with muscle degeneration.

## 2. History of Chimerism and the Development of Hematopoietic Chimeric Cells

### 2.1. Chimerism Promotes Tolerance Induction

Chimerism is characterized by the presence of donor-derived cells within the organism of a genetically distinct recipient which do not induce an immunological response [35,36]. In humans, chimerism occurs naturally during pregnancy due to the migration of fetal cells across the placenta into the maternal circulation, or the opposite occurs, where maternal cells are transferred into the fetus. These cells may travel into the mother’s or fetus’s bloodstream, migrate to different organs, and may remain in the mother’s body or the child’s body for a decade after childbirth [37]. Chimerism can also occur after blood transfusion or stem cell or bone marrow transplant following the transplantation of organs and tissues between genetically different individuals [38].

The concept of chimerism first emerged in the early 1950s and was initially conducted by an intravenous injection of adult donor bone marrow and spleen cells into fetal and neonatal mice (within 24 h after birth) [39,40]. These studies described the induction of the transplantation tolerance expressed as an absence of an immunological response to a skin allograft in the recipients, adult mice. Subsequent publications explored tolerance induction through mixed chimerism [41,42], aiming to prevent the development of Graft-versus-Host Disease (GvHD) following transplantation. Moreover, the number of studies on animal models showed that the infusion of BMC derived from the donor and the depletion of the recipient’s T cells can significantly prolong the allograft survival and reduce the need for immunosuppressive medications [43,44].

The pioneering attempt using donor BMC in a clinical setting was conducted by Monaco et al. in kidney transplant patients [45] and in the first randomized trial in liver transplant patients in 1977 [46]. Furthermore, the study by Scandling et al. established mixed chimerism and induced tolerance towards the kidney allograft, leading to the discontinuation of all immunosuppressive medications six months post-transplant [47,48]. Clinical investigations have stressed that the crucial prerequisite for developing donor chimerism is the migration of passenger leukocytes. This phenomenon has been confirmed in kidney transplants, with the presence of donor-derived cells observed in various recipient organs, including the skin, lymph nodes, peripheral blood, and bone marrow [49,50]. To further assess the immunomodulatory character of hematopoietic cells and their role in chimerism induction, the in vivo creation of a primary and secondary chimera was conducted and confirmed the development of chimerism by the presence of donor and recipient MHC antigens [51].

Based on the existing research on stem cells, the in vivo creation of chimeric cells can be explained by processes such as differentiation, transdifferentiation, cell fusion (CF) [52], and cell maturation surface antigen transfer, known as trogocytosis [53]. The latter two appear to have the most significant impact on chimeric cell creation. Trogocytosis was found to have the ability to modify the cells’ phenotype and alter their immune function, thereby generating immune plasticity beyond genetic and epigenetic programming [54,55,56]. CF is a biological process in which two or more cells create mono- or multinucleated cell hybrids by merging their membranes and contents [57,58,59], allowing them to present a phenotype of undifferentiated cells or possess features of both cell types involved in the fusion [60,61]. The CF is widely used in the production of monoclonal antibodies using hybridoma technology, in which antibodies releasing B lymphocytes are fused with myeloma cells [62]. Moreover, in vivo studies involving bone marrow-derived cells and various cells predisposed to the fusion have proven that the fused cells not only demonstrate the mixed phenotype, but can also take over the function of the damaged recipient cells [63,64,65,66,67].

While these findings provide promising evidence for introducing chimerism as a novel approach to tolerance induction, further progress in cellular-based therapies is imperative to improve the maintenance of HLA-mismatched grafts and to integrate them into routine clinical practice.

### 2.2. Bone Marrow-Based Chimeric Cells

The introduction of clinical BMT dates back to 1957 [68]. Despite the recent advancements, the continuous challenges such as the requirement for lifelong immunosuppression and the occurrence of GvHD highlight the pressing need for innovative approaches to improve patient outcomes and minimize complications. Addressing these limitations demands the development of tailored cellular therapy capable of achieving mixed hematopoietic chimerism without the need for recipient conditioning and lifelong immunosuppression. In response to these challenges, the creation of tailored cellular therapy, known as DRCC, derived from both the bone marrow cells of the transplant donor and the recipient, has emerged as a promising approach [8]. This innovative method involved the initial harvesting of the bone marrow and the isolation of stem cells followed by the creation of the human hematopoietic chimeric cell (HHCC) line from BM-derived CD34^+^ cells, originating from two unrelated human donors [19]. Subsequent in vitro assessments of the HHCC have successfully demonstrated the viability, genotype, phenotype, as well as clonogenic and tolerogenic properties of the new HHCC line, thereby presenting new therapeutic options for hematologic disorders and in transplantation applications.

### 2.3. Umbilical Cord Blood-Based Chimeric Cells

Expanding upon the favorable outcomes observed with bone marrow-based chimeric cell lines, there is scientific evidence of the significant role of umbilical cord blood (UCB) cells as a viable option for tolerance induction in solid organ transplantation, BMT, and VCA [69,70,71]. Leveraging this potential, the new lines of the human umbilical di-chimeric cell (HUDC) [20] and human multi-chimeric cell (HMCC) [72] were created from two and three unrelated human UCB donors, respectively. This innovative therapeutic approach is based on the unique immunomodulatory properties of UCB cells, offering a promising solution for a closer donor-specific HLA-match. Following the confirmation of fusion feasibility, in vitro assessments of HUDC and HMCC confirmed the chimeric state, viability, genotype, hematopoietic phenotype, as well as clonogenic and tolerogenic properties of both chimeric cell lines. The creation of HUDC and HMCC represents a significant achievement in the ongoing effort to develop personalized cellular therapies, offering the potential to enhance patients’ outcomes and reduce treatment-associated burdens in the fields of regenerative medicine and transplant surgery [20,72].

## 3. Chimeric Cells for Treatment of Duchenne Muscular Dystrophy

Duchenne muscular dystrophy (DMD) is a lethal, X-linked disease caused by a mutation in the dystrophin gene, affecting 1 in 3500 to 5000 newborn males per year [73]. Despite extensive research efforts and numerous pre-clinical and clinical studies, DMD still remains an untreatable condition, leading to premature death in adolescent boys due to cardiopulmonary complications [74,75]. Several gene therapies, such as exon skipping [76,77], genome editing using a CRISPR/Cas9 (clustered regularly interspaced palindromic repeats) system [78,79,80], and micro-dystrophin gene delivery via adeno-associated viruses (AAV) [81,82], have been investigated; however, the adverse immune responses, potential for off-target mutations, and the tumorigenicity limit their clinical applications [83]. In contrast, DEC therapy does not rely on the use of viral vectors, reducing the risk of sensitization, and does not require genetic manipulations, which eliminates the possibility of the off-target mutations. Furthermore, therapies based on stem cells, both the autologous and allogeneic cells, have been tested in recent years as one of the most promising approaches for a safe and efficacious strategy for the treatment of DMD [34,84]. Strategies focusing on the autologous cells, harvested from the DMD-affected patients, aim to restore functional dystrophin in the damaged muscles. These options include: mesenchymal stem cells (MSC), bone marrow-derived cells, as well as myoblasts, mesoangioblasts, and cardiomyocytes [18,85,86,87,88,89]. Allogeneic cell lines, such as satellite cells, muscle-derived stem cells, induced Pluripotent Stem Cells (iPSC), mesenchymal stem cells of bone marrow and umbilical cord blood, or cells of adipose tissue origin are also tested for the potential to increase dystrophin expression [33,90,91]. However, due to limitations associated with the rejection of allogeneic cells and the limited engraftment [30,34], it has become critical to develop novel therapeutic strategies that could significantly extend the lifespan and improve the quality of life of DMD patients. The introduction of DEC therapy responded to these unmet needs as evidenced by the increased dystrophin expression correlating with the enhancement of functional outcomes. In addition, the long-term engraftment and tolerogenic properties of DEC cells were confirmed in both animal models and clinical studies, which are summarized below.

### 3.1. Creation of Murine Dystrophin-Expressing Chimeric (DEC) Cells

Since the main challenge of developing an effective cellular therapy with the potential to cure DMD was to overcome the low efficacy of cell engraftment and the requirement for harmful immunosuppression, the unique immunomodulatory properties of chimeric cells have proved to be valuable and are considered as the potential treatment option. The ex vivo-fused DRCC of bone marrow origin possessed tolerogenic properties that improved the maintenance of the engraftment and long-term allograft survival [2,8,16]. Based on the encouraging reports on the application of DRCC, new generations of chimeric cells were created, including several approaches for the development of DEC therapies of myoblast and mesenchymal stem cell origins [17,18]. As a result, the engraftment potential and efficacy of DEC therapies was confirmed following different administration routes [17,18,92,93,94]. The summary of the published work is provided in chronological order and divided into pre-clinical and clinical studies in Table 1.

In 2018, a study on the direct intramuscular administration of DEC cells into the gastrocnemius muscle (GM) of an *mdx* mouse model of DMD was published [17]. The study tested the feasibility and efficacy of DEC therapy created by the fusion of normal and dystrophin-deficient myoblasts (MB*^wt^*/MB*^mdx^*) using polyethylene glycol (PEG). The efficacy of CF and engraftment was confirmed by improvement in muscle strength and function which correlated with increased dystrophin expression in the GM of dystrophin-deficient mice at 30 days following DEC transplantation [17]. The outcome of this study, although encouraging, presented only a local effect in the injected GM. Therefore, to target muscles most severely affected by DMD, including cardiac, diaphragm, and gastrocnemius muscles, the systemic delivery routes were tested, including intraosseous administration, which was proven to be efficient in the delivery of bone marrow and other chimeric cell lines [2]. Therefore, the systemic–intraosseous administration was applied to the delivery of DEC cells of myoblast and MSC origins (MB*^w^*^t^/MB*^mdx^* and MB*^wt^*/MSC*^mdx^*) and confirmed the systemic protective effect on cardiac muscle [95], as evidenced by an increased ejection fraction (EF) and fractional shortening (FS) on an echocardiography (ECHO) 90 days after intraosseous DEC administration. Interestingly, there was also a rebound effect observed between day 30 and 90 after DEC transplant, suggesting an improvement in cardiac contractility correlating with the ECHO-confirmed function [95]. The systemic effect of intraosseous DEC delivery of myoblast and MSC origins was further confirmed by the improved skeletal muscle force and reduced muscle fatigue [94]. Therefore, it was confirmed that DEC cells demonstrate high engraftment potential, corresponding with increased dystrophin expression and significantly improved muscle function following the systemic–intraosseous administration of DEC therapy.

### 3.2. Creation of Human Dystrophin-Expressing Chimeric (DEC) Cells

**Figure 2:**
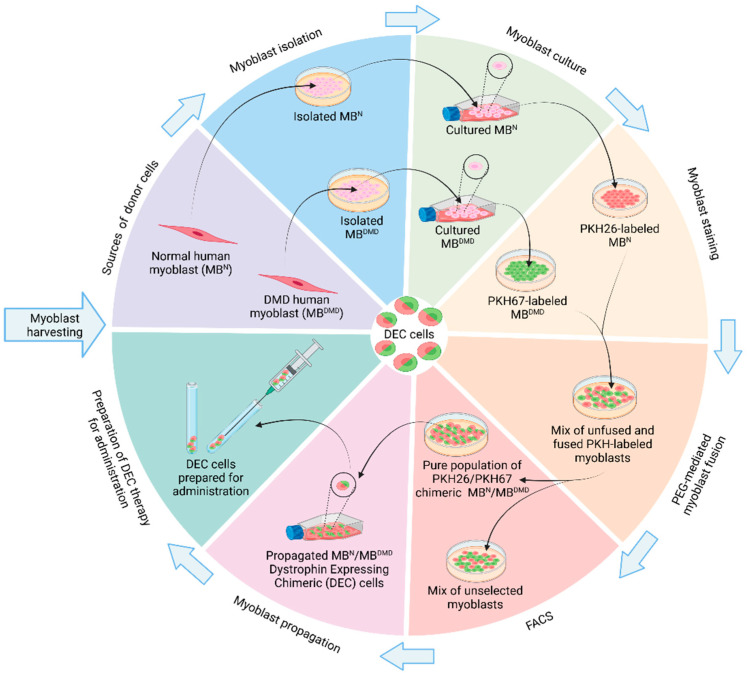
Manufacturing process of Dystrophin-Expressing Chimeric (DEC) cell therapy. The creation of DEC cells starts with muscle biopsies harvested from DMD-affected and normal human donors, proceeded by myoblast isolation and culture, PKH staining, and ex vivo polyethylene glycol (PEG)-mediated fusion, followed by cell sorting via FACS (fluorescence-activated cell sorting) to select pure population of DEC cells for further propagation by preparation of DEC cells for administration. Figure created with BioRender.com.

Promising results of the murine DEC therapy administration encouraged further studies to test human cells in the *mdx*/*scid* mouse model of DMD. Initially, the creation of human DEC cell lines was conducted by the ex vivo fusion of normal human myoblasts from two healthy donors (MB^N1^/MB^N2^) and from normal and DMD-affected donors (MB^N^/MB^DMD^) (Figure 2). These two new human DEC lines were tested in the *mdx*/*scid* mouse model of DMD and confirmed increased dystrophin expression correlating with improved muscle strength and function assessed by a standard functional test at 90 days following a local intramuscular injection [21].

In addition to myoblast-based human DEC lines, due to the immunomodulatory role of MSC, Siemionow’s team created chimeric cells of human myoblast (MB) and MSC origins derived from normal healthy donors (MB^N^/MSC^N^) [18]. MSCs have been previously used in clinical trials involving DMD patients [87] as their ability for rapid proliferation, potential for myogenic conversion, and immunomodulatory properties were considered to be beneficial for muscle regeneration. Meanwhile, myoblasts are valued for their dystrophin delivery potential. Hence, the new human MB^N^/MSC^N^ line was proven to be safe by a COMET assay and by a reduced allogeneic immune response when injected into the GM of an *mdx*/*scid* mouse model of DMD [18]. However, due to the higher dystrophin expression observed after the administration of human DEC cells of a myoblast origin, when compared to MSC-based therapy, the myoblast-based DEC cell line was found to be more beneficial for application in DMD patients.

As previously discussed, the primary advantage of the intraosseous delivery of DEC cells lies in the ability to generate systemic effects on various DMD-affected muscles. Moreover, the intraosseous administration of DEC cells allows the bypassing of the “first-pass“ effect, thereby preventing the entrapment of cells in the lungs, liver, or spleen, which is an issue commonly observed with intravenous cell infusions, such as those involving MSCs. It should also be emphasized that an intraosseous injection is a much shorter and straightforward procedure than intramuscular delivery, which takes several hours and requires anesthesia. Therefore, the intraosseous administration of chimeric cell therapies including DEC was found to be a preferable route of delivery, specifically more optimal for clinical application in the pediatric population of DMD patients.

With this consideration, as the next step in the development of DEC cell technology for DMD and muscle regeneration, the systemic–intraosseous administration of human DEC cells derived from normal and DMD-affected donors following delivery to the *mdx*/*scid* mouse model of DMD was tested [92,93,96,97]. These studies confirmed a long-term DEC cell engraftment, with a significant increase in dystrophin expression correlating with functional improvements in cardiac, pulmonary, and skeletal muscle function confirmed by echocardiography, plethysmography, and standard muscle strength tests [92,93]. The additional benefit observed after systemic DEC therapy delivery was a significant improvement in *mdx* muscle pathology, expressed by reduced muscle fibrosis and inflammation, a decreased number of centrally nucleated fibers, and an overall normalization of myofibers’ morphology [92,93].

Since all new therapeutic strategies have to be tested for safety, the safety of the myoblast fusion procedure and the biodistribution of created human DEC cells to the target and non-target organs were further assessed. The fusion safety was confirmed by the lack of DNA damage, whereas the biodistribution studies confirmed the absence of tumorigenicity by MRI and the preferential distribution of DEC cells to the DMD-affected target organs of the heart, diaphragm, and extremity muscles. Moreover, the human origin of engrafted cells was confirmed by the presence of the human HLA-ABC antibodies’ expression in the tested muscles. Importantly, there was a negligible presence of the cells in the non-target organs, including the lungs, liver, and kidneys, confirming the long-term safety of DEC therapy after intraosseous administration. These studies confirmed the trafficking of human DEC cells from the injection site of the bone marrow compartment to the target, DMD-affected organs [96]. The migration of DEC cells from the bone marrow to the bloodstream via the capillary system is mediated by signals, such as inflammatory cytokines, chemokines, and other factors released by the damaged muscle tissue. Once mobilized, DEC cells enter the circulation and after receiving signals from the target organs, are guided to the damaged tissue sites. Subsequently, DEC cells engraft within the muscle fibers and contribute to the muscle repair and regeneration process. A similar pattern of migration was reported for MSCs and hematopoietic stem cells [101,102].

The promising outcomes of these preclinical studies introduced human DEC cell lines as a novel therapeutic strategy for DMD, demonstrating the potential of DEC cells to both halt the disease’s progression and significantly improve the function of the affected muscles. The most essential quality of DEC therapy is that DEC cell creation does not require cellular reprogramming, genome-editing, or viral vector-induced engineering, and has proven to be safe. Consequently, these encouraging results of the preclinical phase of DEC therapy development support the first in-human study [98,99,100].

### 3.3. Dystrophin-Expressing Chimeric (DEC) Cell Therapy in the First in-Human Study

The introduction of DEC cells to the clinical studies represented the next important step in the development of myoblast-based chimeric cell technology. The primary goal of the first in-human study, initiated in 2021, was to assess the safety and efficacy of a single dose of DT-DEC01 therapy, administered to 6–15 years old boys with genetically confirmed DMD. The study was designed as a single-site, open-label pilot study for the enrollment of ten DMD patients, regardless of the gene mutation and the ambulatory status [98,99,100]. Study participants received a single dose of (2 × 10^6^/kg body weight) DT-DEC01 cells via direct intraosseous administration to the bone marrow cavity of the iliac crests. Following administration, the participants were subjected to a 6-month active follow-up period, and 18 months of passive follow-up [98,99,100].

The personalized DT-DEC01 therapy was created by the fusion of myoblasts of DMD patients and normal donors (typically, the patient’s father or close relative) obtained from an open muscle biopsy. Blood samples were taken from DMD patients and the normal donors for HLA typing, and the patient’s sera were assessed for the presence of the anti-HLA antibodies and donor-specific antibodies (DSA) [98]. Vital signs and the laboratory tests of the participants were monitored throughout the entire study and the follow-up period to assess safety, while functional tests were performed to evaluate the efficacy of the DT-DEC01 therapy. The functional tests conducted in ambulatory patients included the 6-Minute Walk Test (6MWT) and timed tests of the NorthStar Ambulatory Assessment (NSAA). All participants, ambulatory and non-ambulatory, underwent ECHO [98,99,100]. Moreover, the hand grip was evaluated by a dynamometer, the Performance of Upper Limb (PUL 2.0) test assessed the upper limb function, and an electromyography (EMG) assessed the Motor Unit Potential (MUP) duration [98,99,100], while spirometry was assessed in the non-ambulatory patient. All participants had their activity monitored with the step or arm movement counter (Vivosmart 4, Garmin, Southampton, UK) [98,99,100].

At the time of this review, three articles describing the outcomes of the study were published and the authors reported no treatment-related Adverse Events (AEs) or Serious Adverse Events (SAEs) and no presence of the DSA antibodies, thus confirming the safety and tolerability of DT-DEC01 therapy without the need for immunosuppressive therapy [98,99,100]. Furthermore, the authors confirmed the efficacy of DT-DEC01 therapy, revealed by the improvements in the functional tests during the first 6 months after therapy administration, followed by either further functional improvements or the maintenance of the improved parameters at the 12-month follow-up, indicating the halting of disease progression above the baseline levels [98,99]. The most notable outcomes were observed in the maintenance of the cardiac parameters values of EF and FS at the baseline level at 12 months following the intraosseous administration of the single dose of DT-DEC01. This is a significant and clinically relevant finding, considering the progressive nature of cardiac muscle degeneration, responsible for the heart failure, which is the main cause of mortality of DMD patients. Interestingly, the cardiac improvements are correlated with the spirometry results, revealing increased FVC (forced vital capacity) and FEV1 (forced expiratory volume in the first second) values in non-amublatory patients, which are of clinical significance [98,99].

The authors have drawn special attention to the fact that improvements in functional tests correlated with improved EMG parameters and an increased activity level recorded for all patients, as measured by the designated wristband counter of daily steps and arm movements in ambulatory and non-ambulatory patients, respectively [98,99,100]. The functionality of EMG as a biomarker for muscle function monitoring was described in detail in the study published by Niezgoda et al. [100] and confirmed its reliability by an increase in MUP duration, amplitudes, and polyphasic MUPs, which correlated with an improvement in muscle function.

The first in-human study testing the safety and efficacy of DT-DEC01 therapy after the systemic–intraosseous administration of a single dose to DMD-affected patients is still in progress. However, the results achieved so far support DT-DEC01 therapy as a novel therapeutic strategy for DMD which does not require genetic modifications, viral vectors, or immunosuppression and, therefore, can be safely administered clinically [98,99,100].

Finally, the most significant feature and novelty of DT-DEC01 which has to be emphasized is that it does not depend on the type of gene mutation, viral vectors manipulation, or the ambulatory status of the patients, and as such, introduces DT-DEC01 as a universal therapy for all DMD patients and other muscle dystrophies.

## 4. Future Potential of Chimeric Cells and Other Stem Cell Therapies in Treatment of Different Muscular and Neuromuscular Disorders

Based on the encouraging outcomes of DEC therapy administration in both preclinical and clinical studies [17,18,21,92,93,94,95,96,97,98,99,100], it is reasonable to suggest that DEC cells could provide potential therapeutic benefits in other muscular dystrophies and neuromuscular disorders. The unique qualities of DEC cells, such as the tolerogenic and immunomodulatory properties, the potential to ameliorate fibrotic and inflammatory muscle changes, and the positive mitochondrial effect, could contribute to the treatment of other rare disorders and promote muscle regeneration and improvement of function.

### 4.1. Regenerative Properties of DEC Cells as the Potential Treatment of Other Rare Muscular Dystrophies

Considering that DMD represents one of the many muscular dystrophies, it is expected that human DEC therapy would have beneficial effects in the treatment of other types of muscular genetic disorders. Becker Muscular Dystrophy (BMD) is the second most common type of muscular dystrophy after DMD; however, it is generally less severe and has a later onset [103]. BMD is characterized by mutations that partially reduce the production of dystrophin, rather than eliminating it completely [104]. Nevertheless, given that DEC cells carry the potential to increase dystrophin expression, the application of DEC therapy could provide a source of this crucial protein for muscle regeneration. Consequently, this approach could yield benefits similar to those observed after the administration of DEC therapy in the *mdx* animal models of DMD and in the clinical trials, where the administration of DT-DEC01 therapy resulted in an improvement in muscle strength and function correlating with the halting of disease progression [98,99,100].

There are other types of non-dystrophin-deficient, rare muscular dystrophies, including Facioscapulohumeral Muscular Dystrophy (FSHD), Limb-Girdle Muscular Dystrophy, or Congenital Muscular Dystrophy (CMD). Patients with these dystrophies could benefit from DEC therapy, since currently there are no therapies available for these rare muscular disorders. A common characteristic of all types of muscular dystrophies is progressive muscle weakness and degeneration [105,106,107]. Therefore, enhancing muscle regeneration and minimizing muscle atrophy by reducing muscle inflammation and fibrosis and improving muscle fibers’ morphology would be critical for the enhancement of muscle regeneration and improvement of function. The administration of DT-DEC01 resulted in an improvement in the clinical outcomes in DMD [98,99,100]; therefore, the application of DEC therapy could have beneficial effects in the treatment of other rare muscular dystrophies.

### 4.2. Immunomodulatory and Anti-Inflammatory Properties of MSCs and Myoblast-Based DEC Cells as the Potential Therapeutic Strategy for Autoimmune Disorders Involving Muscle Degeneration

There are several known autoimmune disorders which target skeletal muscles and lead to muscle degeneration. The inflammatory myopathies, such as polymyositis and dermatomyositis, are characterized by muscle inflammation leading to muscle weakness [108,109]. In polymyositis, muscle inflammation is triggered by the cytotoxic T cell recognition of an unidentified autoantigen [110]. Patients with this condition commonly present with muscle weakness, particularly affecting the proximal musculature, along with the flexion of the neck and torso [111]. In dermatomyositis, which may be triggered by cancer or a viral infection, muscle weakness symptoms are accompanied by a skin rash [112,113]. The treatment options for these two autoimmune disorders primarily rely on steroids or other immunosuppressive regimens, which can potentially induce severe side effects [114,115]. Therefore, the potential clinical benefits of MSCs in the treatment of autoimmune diseases have been explored in numerous studies involving animal models. However, only sporadic clinical trials showed encouraging outcomes [116]. Despite improvements in serological markers and muscle strength after intravenous MSC infusion, prospective trials were recommended to assess the long-term efficacy [117]. DEC therapy based on MSCs, known for their anti-inflammatory and immunomodulatory properties, was previously tested in animal models [18,95]. However, human DEC therapy of a myoblast origin revealed higher levels of dystrophin expression combined with tolerogenic and anti-inflammatory properties and, therefore, could provide a positive effect on inflammatory changes observed in muscles affected by polymyositis or dermatomyositis.

### 4.3. Therapeutic Potential of Stem-Cell-Based Therapies and DEC Therapy as a Novel Approach for Metabolic Muscle Disorders

Fabry disease, a lysosomal storage disorder, arises from the deficiency of α-galactosidase A (α-GalA), leading to the accumulation of toxic metabolites such as globotriaosylceramide (Gb3) and globotriaosylsphingosine (lysoGb3) [118]. Patients diagnosed with Fabry disease typically report muscle pain, especially during physical activity, as well as with fatigue and asthenia [119]. Additionally, exercise intolerance and muscle fatigue may arise from heart failure and diastolic dysfunction, contributing to secondary skeletal muscle abnormalities [120]. Furthermore, lipid accumulation in various cell types manifests also through other symptoms, including angiokeratoma, corneal opacity (cornea verticillata), neuropathic pain (acroparesthesias), heat intolerance, anhidrosis, microalbuminuria, progressive kidney disease, and cerebrovascular complications such as stroke [121]. Therapeutic options remain limited, primarily revolving around enzyme replacement therapy (ERT) and oral pharmacological chaperone therapy (PCT) [122]. Therefore, the anti-inflammatory and adipose tissue-reducing properties of DEC therapy may offer benefits for patients with Fabry disease, potentially reducing the accumulation of Gb3 and lysoGb3. Another lysosomal disorder is Pompe disease, a rare autosomal recessive neuromuscular condition that can manifest across all age groups, characterized by a deficiency of the acid alpha-glucosidase (GAA) enzyme [123]. This deficiency leads to the accumulation of lysosomal glycogen in various tissues, including skeletal, cardiac, and smooth muscle tissues [124]. Pompe disease predominantly impacts the muscles of the pelvic region rather than those of the shoulder girdle, with scapular winging often prominently observed [125]. Furthermore, patients commonly present with complaints of exercise intolerance, fatigue, or myalgia, which may progress to limb-girdle and axial weakness, ultimately culminating in respiratory failure [126]. Although ERT remains the standard of care for Pompe disease, it does not halt its progression and its long-term efficacy remains uncertain [125].

Alternatively, novel therapies based on genetically modified hematopoietic stem/progenitor cells (HSPC) have been introduced for managing certain metabolic diseases, including lysosomal storage disorders [127,128,129]. These potential therapeutic approaches tested in Fabry and Pompe diseases use lentiviral vectors to increase the expression of α-GalA and GAA, respectively [127,130]. However, one of the major concerns associated with gene delivery was mutagenesis and genotoxicity. Furthermore, the harvesting of HSPC requires host myeloablation which leads to severe adverse effects and achieving the long-term engraftment of infused cells remains a significant challenge [131]. Therefore, DEC therapy could be considered as an alternative therapeutic approach for lysosomal storage disorders. The delivery of DEC cells does not require viral vector-induced engineering, thus eliminating the risk of off-target mutations. Additionally, the anti-inflammatory and adipose tissue-reducing properties of DEC therapy could offer therapeutic benefits in these diseases.

Other metabolic muscle disorders with limited treatment options include McArdle’s disease and Danon disease. McArdle’s disease is a type of glycogen storage disease that arises from a deficiency of myophosphorylase, an autosomal recessive genetic disorder primarily affecting skeletal muscles [132]. The absence of the myophosphorylase enzyme results in glycogen accumulation within tissues, leading to symptoms such as exercise intolerance, muscle cramps, and myoglobinuria following physical exertion [133]. Patients typically present with painful muscle cramps, weakness, and fatigue, which manifests during periods of physical activity [134]. Therapeutic options are currently limited, with dietary interventions showing efficacy in ameliorating clinical manifestations [135]. Danon disease is an X-linked dominant disorder primarily impacting skeletal and cardiac muscles [136]. This condition affects males more commonly and is characterized by cardiomyopathy, skeletal myopathy, and varying degrees of intellectual disability [137]. Patients commonly present with muscle weakness and potential delays in motor skills development. Typically, larger muscle groups such as those of the back, shoulders, neck, and upper legs are affected [138]. Symptoms may manifest as difficulty in raising the arms, rising from a seated position, or climbing stairs. The presence of fatigue, dyspnea, and lower extremity edema could indicate progressive cardiomyopathy [139]. It stems from genetic anomalies in the lysosome-associated membrane protein 2 (LAMP2) gene, responsible for encoding the LAMP2 protein [139]. Defects in this gene result in the accumulation of autophagic material, often accompanied by glycogen, within skeletal and cardiac muscle cells [140].

Therapeutic options for both conditions are limited, with dietary interventions showing some efficacy in reducing clinical manifestations [135]. To date, no cell-based therapies have been approved for the treatment of McArdle’s disease or Danon disease. However, a preclinical model using human iPSCs for McArdle’s disease was tested as a platform to test drugs or compounds with potential pharmacological activity [141]. A similar approach was explored for Danon disease to develop iPSC-based models for potential therapeutic applications [142]. Moreover, genetically modified hematopoietic stem cell-based therapies are currently tested in preclinical studies, but their efficacy has yet to be established [143]. The primary safety concerns for these therapies are the risks of tumorigenicity and immunogenicity. Therefore, DEC could be considered as a new therapeutic option for these diseases since the DEC safety profile been already been established in numerous studies. Additionally, the immunomodulatory properties of DEC cells and protective effect on muscle morphology could lead to the mitigation of muscle weakness, and increased tolerance to the accumulated products including autophagic material and glycogen.

### 4.4. Unique Properties of Stem Cells and DEC Cells as the Supportive Therapy for Neuromuscular Disorders

Neuromuscular disorders are a broad group of diseases that affect the peripheral nervous system. One of the rare but severe conditions is Amyotrophic Lateral Sclerosis (ALS), a fatal progressive disease that affects motor neurons in the brain and spinal cord [144,145]. Patients with ALS experience muscle weakness caused by muscle atrophy, which is accompanied by frontotemporal dementia due to degeneration affecting both upper and lower motor neurons [146]. The precise mechanisms underlying motor neuron death remain poorly understood; however, it is believed to combine both genetic and environmental factors [147]. Moreover, mitochondria play a critical role in the pathology of ALS as their functions, such as energy production, calcium regulation, and apoptotic signaling, are crucial for neuron survival [148]. Currently, there is no cure for this devastating disease and management primarily revolves around palliative care [149,150]. Therefore, there is an urgent imperative to explore new and effective therapies.

Another rare neuromuscular disorder is spinal muscular atrophy (SMA), caused by genetic mutations in the SMN1 gene resulting in progressive motor neuron degeneration [151]. Symptoms manifest earlier in life, compared to ALS, and include muscle wasting and weakness [152,153]. While the introduction of nusinersen marked a breakthrough in SMA management, access to this therapy still remains restricted [154,155].

Many researchers are exploring the potential of iPSCs in disease modeling to generate different cell phenotypes, which could contribute to the development of personalized therapies for a variety of neurodegenerative disorders [156,157]. Moreover, embryonic and neural stem cells have been expanded in the preclinical setting for similar purposes [158]. However, these approaches are challenged by the potential for the off-site mutations and tumorigenicity. Therefore, other approaches, including DEC cells, could offer benefits as supportive therapy for neuromuscular disorders. Specifically, the potential of DEC cells to deliver healthy mitochondria, normalize fiber size morphology, and reduce the inflammation and fibrosis of the affected muscles could lead to improvements in muscle-associated symptoms in ALS and SMA.

### 4.5. Promising Prospects of Mesenchymal Muscle Stem Cells and DEC Therapy in Addressing Multifactorial Pathophysiology of Sarcopenia

Considering all the aforementioned conditions, sarcopenia emerges as the condition most likely to benefit from DEC therapy application. Sarcopenia is characterized by generalized muscle loss resulting from aging, immobility, and nutritional deficiencies often seen in cancer patients [159,160]. While several potential factors may contribute to its incidence, the precise cause remains elusive. Nevertheless, a notable change observed in sarcopenic muscles is the redistribution of muscle fibers, with type II muscle fibers (“fast twitch fibers”) being replaced by type I muscle fibers (“slow twitch fibers”) [161,162]. Moreover, pathological changes, including chronic inflammation and adipose tissue infiltration can also be observed in muscles affected by this condition, as well as mitochondrial dysfunction, leading to the reduced production of energy and increased antioxidative stress which plays a significant role in the pathophysiology of sarcopenia [163].

Currently, the most common treatment options for sarcopenia include nutritional support and exercise therapy [164]. However, the frequent and severe nature of this condition has encouraged researchers to explore the potential of stem cell-based therapies in sarcopenia. Satellite cells (SCs) and muscle-derived stem cells were tested for their potential in muscle regeneration, but their application is limited by the difficulty of SCs’ isolation and purification [165]. Additionally, the immunomodulatory properties of MSCs could provide benefits in reducing muscle pathology, even though they do not differentiate into myogenic cells [166]. Therefore, the consideration of DEC therapy, with its known tolerogenic, anti-inflammatory, and anti-fibrotic characteristics, could lead to an improvement in the strength and amelioration of pathology in sarcopenia-induced muscle wasting and degeneration.

## 5. Conclusions

Muscular disorders, such as DMD, represent a significant therapeutic challenge, making the development of new, innovative treatment options critically important. The proposed approaches need to overcome numerous challenges, including immune responses, delivery to all muscle groups, and ensuring the long-term efficacy. The advancements in the field of cell-based strategies introduced chimeric cell therapies with the potential to overcome current challenges and to contribute to the enhancement of muscle repair and regeneration. The tolerogenic and immunomodulatory properties of DRCC lines have demonstrated the potential for long-term cell engraftment and the enhancement of function without the need for lifelong immunosuppression. Furthermore, the novel concept of DEC cells created by the fusion of human myoblasts from normal and DMD-affected donors was successfully tested in preclinical models and in a clinical study.

The major advantages of the newly introduced DEC therapy are that it does not require viral vectors or genetic manipulations, it eliminates the risk of off-target mutations, and is not dependent on the gene mutation, making DEC the first universal therapy for all DMD patients. Moreover, DEC cells are characterized by long-term engraftment and tolerogenic properties, and their safety and efficacy were confirmed in preclinical and clinical studies. The shortcomings of DEC therapy have yet to be established as the clinical trials progress. However, in the future, DEC therapy redosing will be considered to maintain the long-lasting therapeutic effects of DEC cells after administration to the DMD patients.

The results of the first in-human study involving the intraosseous administration of DT-DEC01 therapy have laid the groundwork for addressing other muscular dystrophies and disorders, where current treatment options are limited and new therapeutic strategies are needed. Therefore, chimeric cell technology holds the potential to revolutionize the treatment of DMD and other muscular dystrophies and disorders, where the enhancement of muscle regeneration is crucial for the improvement of muscle strength and function.

The introduction of DT-DEC01 therapy to different clinical applications would have an important impact on the lifespan and quality of life of DMD patients and will play a significant role in treating other muscle degenerative conditions.

## Figures and Tables

**Table 1:** Assessment of creation, efficacy, and safety of Dystrophin-Expressing Chimeric (DEC) cell therapy tested in **A**. pre-clinical animal models of *mdx* and *mdx/scid* mice and **B**. clinical studies involving DMD patients.

A						
Donor	Recipient	Cell Source	Name of the Therapy	Title of the Manuscript	Year	Refs
Mice	*Mdx* mouse	Myoblast	DEC	Creation of Dystrophin Expressing Chimeric Cells of Myoblast Origin as a Novel Stem Cell Based for Duchenne Muscular Dystrophy	2018	[17]
	*Mdx* mouse	Myoblast and MSC	DEC	Cardiac Protection after Systemic Transplant of Dystrophin Expressing Chimeric (DEC) Cells to the *mdx* Mouse Model of Duchenne Muscular Dystrophy	2019	[95]
	*Mdx* mouse	Myoblast and MSC	DEC	Intraosseous Transplant of Dystrophin Expressing Chimeric (DEC) Cells Improves Skeletal Muscle Function in *mdx* Mouse Model of Duchenne Muscular Dystrophy	2022	[94]
Human (Tissues and Cells Bank *)	*Mdx*/*scid* mouse	Myoblast	DEC	Dystrophin Expressing Chimeric (DEC) Human Cells Provide a Potential Therapy for Duchenne Muscular Dystrophy	2018	[21]
	*Mdx*/*scid* mouse	Myoblast and MSC	DEC	Transplantation of Dystrophin Expressing Chimeric Human Cells of Myoblast/Mesenchymal Stem Cell origin Improves Function in Duchenne Muscular Dystrophy	2021	[18]
	*Mdx*/*scid* mouse	Myoblast	DEC	Human Dystrophin Expressing Chimeric (DEC) Cell Therapy Ameliorates Cardiac, Respiratory and Skeletal Muscle’s Function in Duchenne Muscular Dystrophy	2021	[92]
	*Mdx*/*scid* mouse	Myoblast	DEC	Long-Term Protective Effect of Human Dystrophin Expressing Chimeric (DEC) Cell Therapy on Amelioration of Function of Cardiac, Respiratory and Skeletal Muscles in Duchenne Muscular Dystrophy	2022	[93]
	*Mdx*/*scid* mouse	Myoblast	DEC	Long-Term Biodistribution and Safety of Human Dystrophin Expressing Chimeric Cell Therapy After Systemic-Intraosseous Administration to Duchenne Muscular Dystrophy Model	2022	[96]
	*Mdx*/*scid* mouse	Myoblast	DEC	Amelioration of Morphological Pathology in Cardiac, Respiratory, and Skeletal Muscles Following Intraosseous Administration of Human Dystrophin Expressing Chimeric (DEC) Cells in Duchenne Muscular Dystrophy Model	2024	[97]
**B**						
Donor	Recipient	Cell Source	Name of the Therapy	Title of the Manuscript	Year	DOI
Human (normal and DMD-affected donors)	Human	Myoblast	DT-DEC01	Dystrophin Expressing Chimeric (DEC) Cell Therapy for Duchenne Muscular Dystrophy: A First-in-Human Study with Minimum 6 Months Follow-up	2023	[98]
	Human	Myoblast	DT-DEC01	Safety and Efficacy of DT-DEC01 Therapy in Duchenne Muscular Dystrophy Patients: A 12-Month Follow-Up Study After Systemic Intraosseous Administration	2023	[99]
	Human	Myoblast	DT-DEC01	Assessment of Motor Unit Potentials Duration as the Biomarker of DT-DEC01 Cell Therapy Efficacy in Duchenne Muscular Dystrophy Patients up to 12 Months After Systemic-Intraosseous Administration	2023	[100]

* Lonza Bioscience (Mapleton, IL, USA), Axol Bioscience Ltd. (Little Chesterford, UK), and Creative Bioarray Ltd. (Shirley, NY, USA).
